# Keywords and Co-Occurrence Patterns in the Voynich Manuscript: An Information-Theoretic Analysis

**DOI:** 10.1371/journal.pone.0066344

**Published:** 2013-06-21

**Authors:** Marcelo A. Montemurro, Damián H. Zanette

**Affiliations:** 1 Faculty of Life Sciences, The University of Manchester, Manchester, United Kingdom; 2 Consejo Nacional de Investigaciones Cientficas y Técnicas, Centro Atómico Bariloche e Instituto Balseiro, San Carlos de Bariloche, Ro Negro, Argentina; Hungarian Academy of Sciences, Hungary

## Abstract

The Voynich manuscript has remained so far as a mystery for linguists and cryptologists. While the text written on medieval parchment -using an unknown script system- shows basic statistical patterns that bear resemblance to those from real languages, there are features that suggested to some researches that the manuscript was a forgery intended as a hoax. Here we analyse the long-range structure of the manuscript using methods from information theory. We show that the Voynich manuscript presents a complex organization in the distribution of words that is compatible with those found in real language sequences. We are also able to extract some of the most significant semantic word-networks in the text. These results together with some previously known statistical features of the Voynich manuscript, give support to the presence of a genuine message inside the book.

## Introduction

The Voynich manuscript–named after the Polish-American antiquarian Wilfrid Voynich, who owned it since 1912 until his death in 1930-is perhaps the most widely known example of a book written in an as yet undeciphered script. Its author and language are unknown, and no other document in the same script has ever been found. The manuscript‚s ownership history can be traced back to the seventeenth century, but carbon dating of its vellum and stylistic analysis of its illustrations suggest that it was written around the second half of the fifteenth century (Dr. Greg Hodgins, University of Arizona, personal communication). Presently, the book belongs to the Beinecke Rare Book and Manuscript Library of Yale University, where it is identified as Beinecke MS 408. Public-domain electronic images of the full manuscript are deposited in Wikimedia Commons (commons.wikimedia.org/wiki/Voynich
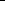
manuscript).

The manuscript comprises 104 folios, organized into 18 quires bound to leather thongs. Both sides of most folios contain text, written from left to right. The text consists of discrete graphemes, chosen from an “alphabet” of some 40 symbols and organized into arrays or “words” of variable length. These arrays are separated by spaces, and lines are sometimes grouped into paragraphs but, otherwise, no evident punctuation marks are used. Most pages also contain illustrations, which modern scholars have used to “thematically” divide the manuscript into five sections: Herbal, Astrological, Biological, Pharmacological, and Recipes. The Herbal section is the longest, and displays dozens of ravishingly coloured plant drawings. Oddly enough, however, not a single one of these pictures could be unquestionably recognized as an existing plant. Similarly, except for the Zodiac signs in the Astrological section, no illustration could be unambiguously interpreted in the whole book.

In spite of its unmistakable medieval-codex look, the origin, purpose, and contents of the Voynich manuscript remain a deep mystery. Since the seventeenth century, numerous attempts at deciphering the script have led to a few claims of success, but none of them has been convincing. Careful quantitative analysis of the text structure, however, has inspired some plausible hypotheses on the manuscrip’s cryptographic nature: while it is unlikely that the book is written in a European language using an unknown alphabetic script, it may be encoding an East Asian language (such as Chinese) into an alphabet invented specifically for such purpose, or contain a more sophisticated encryption of a then familiar language (Latin, for instance). Naturally, the hypothesis of a hoax -a smart fabrication contrived to deceive avid book collectors, of the sort that flourished after the Renaissance times- cannot be discarded either [1], [2].

Transcriptions of the Voynich manuscript script into Roman script, replacing each grapheme of the former by an alphabetic character of the latter, have allowed for the statistical analysis of the text. Most of the studies undertaken in this direction regarded the text as a symbolic sequence of characters (including the blank space). Calculations of the second-order character entropy [3], word statistics and character autocorrelation measures [4], and random-walk-like fluctuations [2], reveal organizational structures that are compatible with a ciphered version of a real language, and make the possibility of a fabrication less likely.

Statistical analyses of the Voynich text regarded as a sequence of space-separated character arrays, or tokens -the manuscript’s “words”- are scarcer, but point toward the same conclusions as the character-based studies [2], [4]. Landini has shown that the collection of tokens satisfies Zipf’s law, with a smooth, approximately inverse relation between the number of occurrences of each token and its rank in a list of tokens ordered by decreasing frequency. In the light of recent research on the emergence of Zipf’s law [5], the probability that the Voynich text resulted from some kind of stochastic process is drastically reduced. Currently, on the other hand, much support is informally given to the hoax hypothesis on the basis that artificial words which are morphologically similar to those of the “Voynichese” language can be created using an adaptation of Renaissance cryptographic techniques [1]. In spite of recurrent claims in that sense, however, not a single piece of quantitative evidence has been advanced showing that such techniques are able to reproduce the features disclosed by statistical analysis of the text structure. It is also worthwhile remarking that the description of such features -which the hoax’s fabricator should have been familiar with in advance-were unquestionably out of reach of sixteenth-century mathematics.

In this study, we present the first analysis of the Voynich manuscript addressing the large scale organizational structure which results from the distribution of words over the whole text. We first apply methods from information theory that identify content-bearing words without any prior knowledge of the underlying language of the text under analysis [6]. Then, we consider putative semantic relationships between the most informative words by analysing their patterns of co-occurrence along the text. This method establishes relationships between the usage of different words along the text, which can be represented by means of semantic networks. A similar procedure is used to determine links between the thematic sections of the text according to their common words.

## Results

Our analysis was performed on a publicly available digital version of the Voynich text in the European Voynich Alphabet (EVA) transcription system. This digital version reproduces the ordering of the manuscript’s current binding, where -except for around a dozen scattered folios- all pages belonging to each “thematic” section are contiguous to each other. Our results were obtained for a reordered version of the manuscript, where those scattered folios were aggregated into the corresponding sections (see Materials and Methods).

### Most Informative Words in the Voynich Text

In any sizable piece of written human language, which articulates information about several subjects, certain words are tightly related to the main topics dealt with in the text. If the Voynich manuscript contains a meaningful text encrypted by translation into a coded or invented language, statistical signatures in the distribution of tokens could be used to identify candidates to play the role of those keywords.

Methods for detecting content-bearing keywords in language samples have a long history [7], [8]. Some of the most successful approaches have looked not only at the frequencies of words, but also at their distribution over the sample [6], [8]–[10]. In particular, the distribution profile of the occurrences of each individual word has turned out to be a key feature to assess the word’s relevance to the overall meaning of a text. While uninformative words tend to have an approximately homogeneous (Poissonian) distribution, the most relevant words are scattered more irregularly, and their occurrences are typically clustered [11]–[13]. The tendency of content-bearing words to cluster over certain parts of the text is a direct consequence of their varying relation to the local semantic context as the text progresses and its meaning unfolds. Over long spans, the clustering patterns of words develop a systematic statistical structure that determines the degree of local specificity of their usage in successive contextual domains. Word clustering has been preliminarily reported in the Voynich text [14].

In our analysis, we used an information-theoretical measure that quantifies the amount of information that the distribution of words bears about the sections where they appear in the text [6]. Words that are uniformly scattered contribute little or no information, since their distribution cannot tag any specific section of the text. On the contrary, words that appear only in certain contextual domains contribute much information, because their distribution identifies those specific sections. The information measure, given by Eq. 2, depends parametrically on a length scale -a given number of words- that defines the size of local domains (see Materials and Methods for an overview).

**Figure 1:**
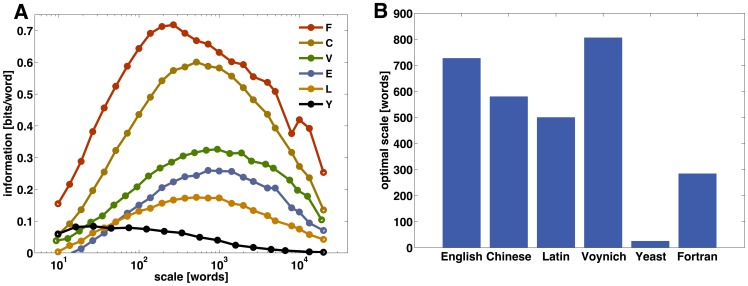
Comparison of the Voynich manuscript and different information carrying sequences. A) Information in word distribution as a function of the scale for the Voynich manuscript compared to other five language and symbolic sequences (F: Fortran; C: Chinese; V: Voynich; E: English; L: Latin; Y: yeast DNA). The number of words in all sequences was equal to that of the Voynich text; if the original sequence was longer, the additional words were not considered. B) Scale of maximal information for the sequences considered in A (see Materials and Methods for more details on the language and symbolic sources).

Figure 1A shows the information in bits per word as a function of the scale of contextual domains for several information-carrying sequences, comprising natural and artificial languages, the Voynich manuscript, and the genetic code (details about the individual sequences are given in Materials and Methods). All cases share a similar overall pattern, with low information for both large and small scales. This feature is a consequence of the fact that, in those two limits, there is poor specificity in the profile of the distribution of words over the text. For sufficiently small scales, all words occur only once or none in each domain, thus making their distribution uninformative about specific locations. In the opposite limit, when the scale becomes comparable to the total length of the text all words have a more or less uniform distribution, which again leads to low information.

In all cases, in turn, the information about the identity of the different local domains attains a maximum at an optimal scale. This is the scale at which the heterogeneity in the distribution of word frequencies over the text is largest. At that particular scale, the frequency profile of the words can, on average, tag different parts most efficiently. For the texts in natural human languages, the maximum achieved by the information varies between approximately 0.2 bits/word for Latin to 0.6 bits/word for Chinese. This difference can be attributed to the disparate size of vocabularies, resulting from the different degrees of inflexion in the respective languages. Latin is a highly inflected language, with nouns and adjectives changing by declension and verbs by conjugation. It typically shows a very rapid growth of vocabulary size as a function of text length [15]. On the contrary, Chinese texts usually require a smaller number of tokens -in this case, characters- for comparable text lengths. Therefore, assuming comparable total information, a smaller effective vocabulary implies more information per word. Interestingly, the three natural language texts attain maximal information at a similar scale of around 600–800 words. The maximal information for the Voynich manuscript is slightly above that of English, and significantly below that of Chinese. Moreover, as can be seen in Figure 1B, the scale at which maximal information is reached for the Voynich text is very similar to that of the human language examples. In contrast, the scales of maximal information for the DNA sequence of the yeast Saccharomyces cerevisiae, and for the Fortran source code are sensibly different from those of the human language texts and the Voynich manuscript. These results suggest that the overall statistics of word distribution over the text in the Voynich manuscript is comparable with that of real human languages.

The total information given by Eq. 2 is a sum of contributions from individual words. It is then possible to assign an information value to each word in the lexicon, corresponding to each term in the sum [6]. This allows a ranking of the individual words according to their contribution to the overall information. In Table 1 we show the 30 most informative words in the Voynich text (in the EVA transcription) ranked by their contribution to the total information, computed both at the optimal scale and with respect to the division of the text in its “thematic” sections. The same procedure applied to texts written in known languages yields a list of keywords that closely relate to their general semantic content [6]. All the words listed in Table 1 have a substantial contribution to the information that their individual distribution profiles bear about the different sections of the text. Despite the fact that the optimal scale is of 807 words while the average size of the “thematic” sections is above 7500 words, there are some words in common in both columns of Table 1, in particular among the most informative. This is because some of these top words in Table 1 are both highly frequent and have a strongly non-uniform distribution over the different “thematic” sections, with some of them being used in only one or two sections of the text. The strong specificity of their distribution is also captured by a partition of the text in sections of equal size, as in the first column of Table 1, thus leading to a high information value.

**Table 1:** The thirty most informative words in the Voynich manuscript.

Optimal Partiton		“Thematic” partition	
shedy	0.00937	daiin	0.00705
qokeedy	0.00840	qokeedy	0.00680
daiin	0.00777	shedy	0.00672
qokaijn	0.00754	chedy	0.00559
chedy	0.00716	chor	0.00512
qokedy	0.00649	qokaijn	0.00487
qokar	0.00538	chol	0.00487
qokeey	0.00518	qokedy	0.00461
chor	0.00514	cthy	0.00456
ol	0.00494	qol	0.00443
chol	0.00458	s	0.00376
s	0.00431	qokeey	0.00339
cthy	0.00431	sho	0.00319
qokaiin	0.00419	ar	0.00313
qokal	0.00372	al	0.00271
al	0.00372	lchedy	0.00263
dy	0.00337	qokaiin	0.00258
ar	0.00327	chy	0.00258
aiin	0.00302	qokal	0.00236
okedy	0.00300	dain	0.00231
okaijn	0.00287	shol	0.00223
lchedy	0.00285	okaijn	0.00221
dain	0.00282	y	0.00200
okeey	0.00281	dy	0.00192
sho	0.00270	qotchy	0.00190
qokain	0.00263	cthol	0.00190
shey	0.00251	shor	0.00189
dal	0.00245	aiin	0.00174
otedy	0.00244	cthor	0.00173
chy	0.00237	qokain	0.00171

The words in the first column corresponds to the ranking obtained for the partition of the Voynich text that renders the highest total information, as given by Eq. 2. In the second column, the partitions used to compute the information associated with each word were the standard “thematic” divisions of the text. For both columns, the numbers to the right are the information values, in bits, contributed by the respective words (see Materials and Methods).

### Affinities between Words and Sections of the Text

By analysing similarities in the patterns of word occurrence, we can establish relationships among words that could be linked by their semantic affinities. Words that are so related will typically tend to co-occur within the same local domains throughout the text.

**Figure 2:**
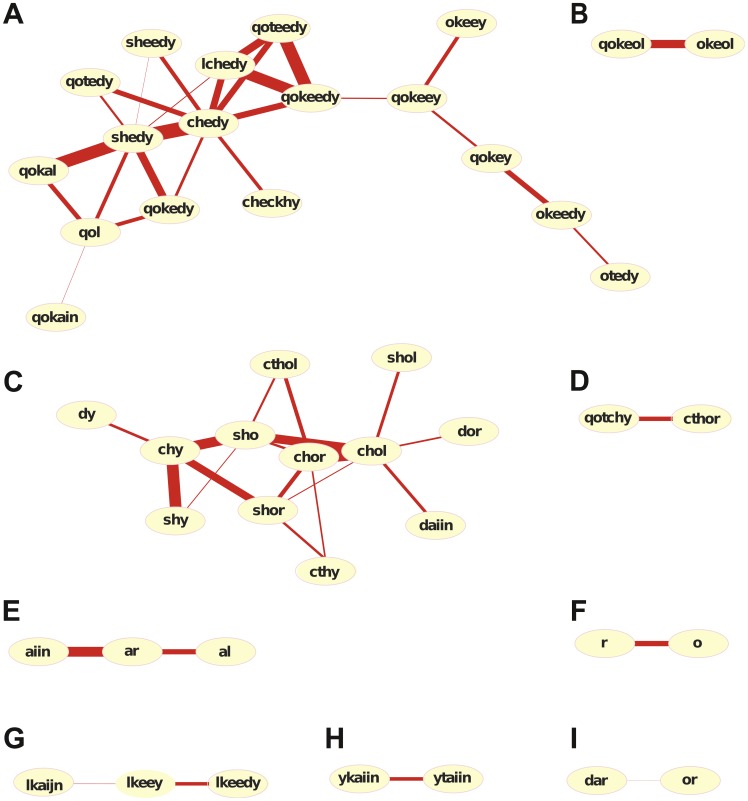
Affinities among the most informative words in the Voynich text. The graph shows words that have similar patterns of occurrence across the text. The analysis was done on the 100 most informative words in the Voynich text. Only words linked by the strongest 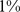 of connections are shown (see text for details).

To group words according to their common patterns of occurrence, we used a vector-space representation of the Voynich lexicon. Each word was associated with a vector whose components were given by its frequency over the different “thematic” parts of the text. We thus built a similarity matrix where the strength of the connection between two words was proportional to their distance in the vector-space, defined by a suitable metric. We performed this analysis on the 100 most informative words of the manuscript. From the resulting similarity matrix we kept the 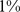 strongest links, and subsequently tested that all those links were statistically significant (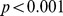, see Materials and Methods). Figure 2 shows the networks determined by the strongest links between words. The thickness of each link indicates the strength of the corresponding connection. The explicit values of the similarity coefficients between pairs of linked words in Figure 2 are listed in Table S1. As a general characteristic for all the clusters shown, the words that are more strongly connected have an evident morphological similarity. Some of the words are linked by their prefixes, as is the case of the strongly connected pair *chol-chor* in the cluster of Figure 2C. In other cases, there is a strong link between words that share a suffix, as with *chedy-shedy* in the group shown in Figure 2A. Taking into account that the clusters were built on the basis of word co-occurrence, and assuming that co-occurrence reveals semantic affinity, we conclude that a strong connection between form and meaning characterizes the most informative words of “Voynichese”.

**Figure 3:**
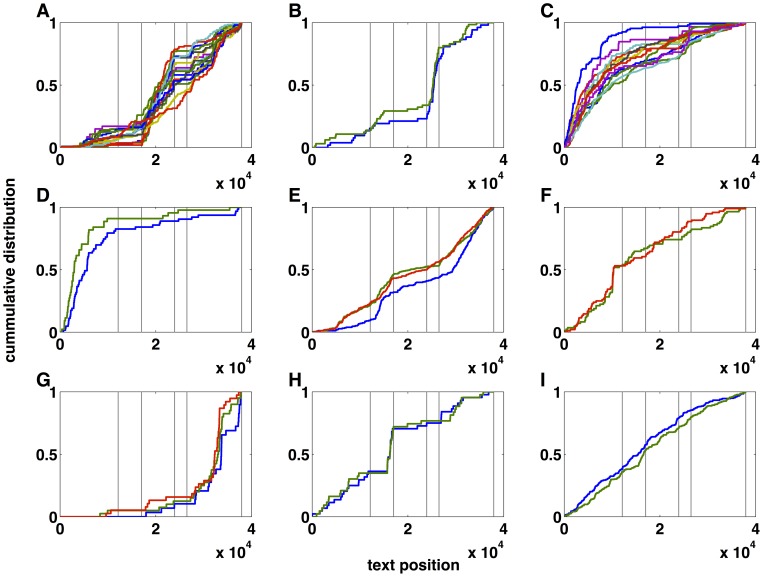
Cumulative distribution of most informative words in the Voynich text. A–I) Cumulative distribution for the same words as in Figure 2. Each panel shows the distribution for the same group of words corresponding to the equally labelled panel in Figure 2. The vertical lines mark the limits of the “thematic” sections of the Voynich manuscript.

The networks shown in Figure 2 link words with similar co-occurrence profiles. Figure 3 shows the cumulative probability of occurrence along the text for each individual word in the networks. In all panels the dotted vertical lines indicate the boundaries of the “thematic” sections of the text. A rapid increase in the cumulative probability of any given word reveals its high frequency in that part of the text, while plateaus are indicative of the word‚s absence there. The degree of specificity of these most informative words with respect to the sections is apparent from the plots. Note, in fact, that sudden changes in the slope of the cumulative probability generally occur at or near section boundaries, which stresses the strong connection between individual words and particular sections. Moreover, it is clear that words within the same cluster have similar occurrence patterns, being specific to the same sections.

**Figure 4:**
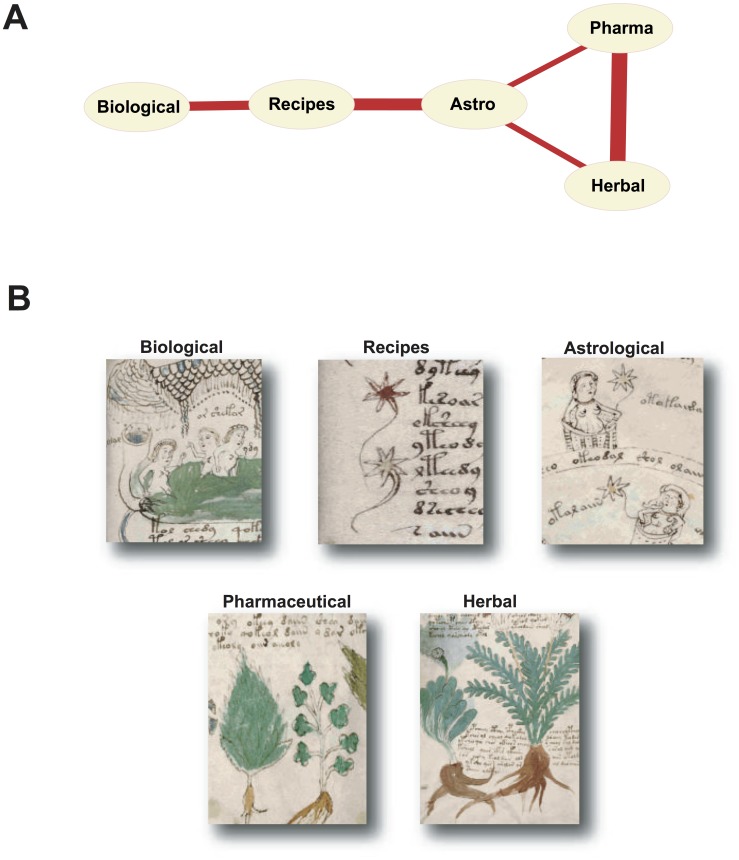
Linguistic and pictorial relationships between the sections of the Voynich manuscript. A) The strongest links between the different sections as determined by the co-occurrence of the most informative words. B) Representative images from the different sections. The two pairs of sections more strongly connected share illustration elements. The Pharmacological and Herbal sections present numerous illustrations of plants, while the Recipes and Astrological sections show numerous instances of flower-like star shapes.

A complementary analysis can be applied to the “thematic” sections of the Voynich manuscript in order to disclose their mutual affinities on the basis of the frequency of the words they contain (see Materials and Methods). Thus, two sections are related to each other if the frequencies of a given set of words are similar in both of them. Figure 4A shows the resulting network of relationships between the sections, while in Figure 4B we present some of the typical illustrations of each section. The values of the similarity coefficients corresponding to the linked sections are listed in Table S2. The strongest link occurs between the Pharmacological and Herbal sections. Inspection of the illustrations from these two sections reveals that they bear a remarkable thematic similarity, being mainly based on the representation of plants. We stress that our analysis establishes, for the first time, a link between these two sections on the sole basis of the linguistic structure of the text. The second strongest link connects the Recipes and Astrological sections. Although these two sections do not share major illustration themes -in fact, the Recipes section contains practically no illustration- it turns out that the characteristic star flower depicted in Figure 4B appears in the Recipes and Astrological sections, and not in others. These results support the conjecture that there is a match between the linguistic structure and the illustrations of the text.

## Discussion

Despite decades of effort, a definite conclusion about the nature of the Voynich manuscript still remains elusive. The hypothesis that it is simply a nonsensical text either intended as a hoax or made up with any other purpose has debilitated in recent years, due to the increasing evidence of the text’s different levels of organizational structure. These regularities, in fact, are compatible with the presence of some kind of linguistic information. Systematic studies supporting the hoax hypothesis have invariably overlooked the fact that any model for the hoax’s fabrication must, at the same time, explain in detail how such linguistic-like structures emerged from the process itself.

One of the strongest clues in this puzzle is the fact that the frequency of words in the Voynich text obeys Zipf’s law [4]. Despite that it has been shown that long random texts exhibit an approximate form of this law, the profile of frequency-rank distributions in human languages differs significantly from that of random symbolic sequences [5], [16]. Precise features of Zipf’s law in languages do not emerge in simple random sequences and generally require interplay between multiplicative and additive processes [15]. Moreover, Zipf’s law was discovered centuries after the accepted date of creation of the Voynich text. Thus, proposed solutions like the use of sixteenth-century cipher methods [1], although not impossible, can hardly account for the presence of Zipf’s law in the Voynich text.

In natural languages, the degree of specificity of words over different parts of a text is determined by their individual semantic role. In particular, content-bearing words appear in texts in a sort of clustered pattern, while structural and functional words tend to be distributed more uniformly [8], [12]. We have analysed the text in the Voynich manuscript using methods derived from Information Theory, that assign a value of information to the individual words in a text without any aprioristic assumption about the structure of the language [6]. Words that are related by their semantic contents tend to co-occur along the text. This property is the basis of standard methods in automatic information retrieval [17]. We compared the patterns of use of the most informative words in the text and found that some of them bear strong relationships in their use. Interestingly, the network of relationships that we obtained showed that related words share similar morphological patterns, either in their prefixes or suffixes. This fact suggests that any underlying code or language in the Voynich manuscript has a strong connection between morphology and semantics, recalling scripts where -as in the cases of Chinese and hierographical Ancient Egyptian- the graphical form of words directly derives from their meaning.

The profiles of word distribution can also be used to find relationships among the different sections of the text. The use of words over the different sections supports a network of relationships between the standard divisions of the text as shown in Figure 4. The sections more strongly linked also show some similarities in the illustration details, thus pointing to consistency in the structure at both the pictorial and textual levels.

In summary, simple methods to generate random texts with some sort of local statistical structure may seem, under superficial scrutiny, rather convincing solutions to the problem presented by the Voynich manuscript. However, the statistical structure of the text at its various levels still requires an explanation that needs to go beyond reproducing local features like word forms or local word sequences. Here, we have contributed evidence of non-trivial statistical structure in the long-range use of words in the Voynich text. While the mystery of origins and meaning of the text still remain to be solved, the accumulated evidence about organization at different levels, limits severely the scope of the hoax hypothesis and suggests the presence of a genuine linguistic structure.

## Materials and Methods

### Electronic Versions of Text and Symbolic Sources

The digital version of the Voynich manuscript is the in the EVA trasncription system was obtained from the site mainatined by J. Stolfi (http://www.ic.unicamp.br/ 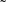
stolfi/voynich/98-12-28-interln16e6/). The other texts used to obtain the results shown in Figure 1 correspond to the following sources: *Confessions* by Augustine of Hippo (Latin), *On the Origin of Species* by Charles Darwin (English), and *The Records of the Grand Historian* by Sima Qian. These texts were downloaded from the Project Gutenberg site (http://www.gutenberg.org/). The Fortran code corresponds to the test driver code for the BLAS (Basic Linear Algebra Subprograms) plus a number of specific routines from the library (http://www.netlib.org/blas/). The file was processed to eliminate all comments, and only commands, variables, and function names were kept. The genetic sequence of the yeast *Saccharomyces cerevisiae* was obtained from the GenBank (http://www.ncbi.nlm.nih.gov/genbank/). Three-nuclei codons were used as word-like tokens for the analysis. To avoid effects due to difference in length, in all cases a section equal in length to the Voynich manuscript was used for the analysis.

### Ordering and Reordering of the Voynich Manuscript

All the manuscript’s folios show numbers, in ordinary European script, on the top right corner of their *recto* side. This consecutive numbering, from 1 to 116, is believed to have been added after the manuscript was bound in its current form. Only 104 folios remain at present. The “thematic” sections of the manuscript have been defined on the basis of their illustrations. Their overall ordering along the text is as follows: Herbal, Astrological, Biological, Pharmacological, and Recipes. While in the present binding of the manuscript most folios belonging to a given section are mutually contiguous, some of them seem to be out of their natural place. The folio numbers corresponding to each section are the following:

Herbal: 1 to 57, 65 to 66, 87, 90, 93 to 96.

Astrological: 67 to 73, 85 to 86.

Biological: 75 to 84.

Pharmacological: 88 to 89, 99 to 102.

Recipes: 58, 103 to 116.

In our analysis of the distribution of words, all the folios of each section have been aggregated into an uninterrupted succession, maintaining their relative ordering inside the section as well as the overall ordering of the sections along the text.

### Information in the Distribution of Words

Here we present an overview of the method used to quantify the information in the distribution of words in a text and to determine which are the keywords with the largest contribution to the overall information. Further details can be found in [6].

Our approach is based on the observation that the most relevant words, or keywords, in a text tend to be more context-dependent than non-informative words. Thus, the specific way in which words are distributed can be used to distinguish statistically different parts of a text. For instance, a word that only appears in one specific chapter of a book is a perfect tag for that chapter, i.e. if that word is encountered, one immediately knows with certainty which chapter is being scanned. Most words will have a less concentrated distribution over the text, but still the non-uniformities in that distribution can associate certain words with specific contextual domains in the text. An objective quantification of that degree of association is given by information theory.

Consider a text of 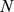 words in length, containing 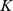 different words. The text is divided into 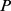 equal parts, of length 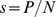. For every word 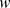 that appears 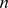 times in the text, we can define its distribution over the text as the probability 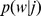 of finding that particular word in part 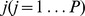. This probability is estimated as the ratio 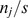, where 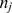 is the number of occurrences of word 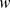 in part 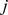, and is normalized as 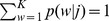. Let us call 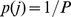 the *a priori* probability that a given word 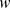 appears in part 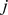, then the overall probability of occurrence of the word is 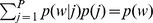, where 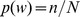. After observing an instance of word 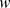, the probability that it comes from part 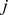 is given by 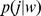, which can be computed as 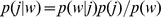, or explicitly in terms of word occurrences as 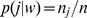.

Then, the mutual information between the sections of the text and the distribution of words is [18]:
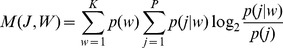
(1)


Words that appear in the text a number of times 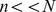 will have statistical fluctuations in their distribution of the parts that may induce an overestimation of the mutual information. We can correct for this bias by subtracting the mutual information computed over randomized versions of the text obtained by shuffling all the words‚ positions. Despite it being computed over random versions of the text, where all the relationships between the words and its original contexts is lost, this quantity will not be zero in general due to the presence of fluctuations in the distribution of words. Let us call 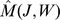 to the mutual information estimated from one realization of the shuffled text. Then, we can define the information in the distribution of words as 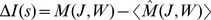, where the average is taken over an infinite number of realizations of the words shuffling. Then, using Eq. 1 and regrouping terms, the expression for 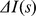 can be written as.
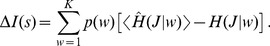
(2)


The entropy quantity 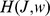 is given by
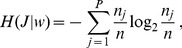
(3)and 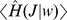 represents the same entropy but computed on a randomly shuffled version of the text and averaged over all possible realizations of the shuffling. This latter expression can be computed analytically [6].

The procedure assigns an information measure to the text that quantifies how much an ideal observer could discriminate between different parts of it just by knowing the distribution of words over sections of a length characterized by the scale. To illustrate the range of entropy values obtained for the words in the Voynich text, Figure S1 shows the estimation of Eq. 3 computed on the original text and on a randomly shuffled version of it, together with the averaged entropy used in Eq. 2.

From Eq. 2 it is apparent that the total information is a sum of contributions from individual words. Each word can be assigned an information measure equal to 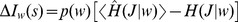. Therefore, the information contributed by individual words depends both on their frequency and on the difference of entropy of computed on the real text and on a random version of it. When words are ranked by their contribution to the overall information in a text, the top words are those more closely related to its semantic content [6].

For the words listed in Table 1, we obtained significance 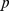-values by means of a bootstrap procedure. The information for each word in the Voynich manuscript was compared with estimates obtained from randomly shuffled versions of the Voynich text. Then, a 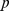 value can be computed as the fraction of the random realizations that yielded a value of the information equal or higher than that measured in the real text. For all the words shown in Table 1, 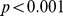.

As an example of the application of Eq. 2, Table S3 shows the top most informative words for three texts in English.

### Word-space Similarity Analysis

Once the most informative words are extracted, it is possible to establish a similarity measure between them by comparing their frequency profiles over the 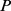 parts of the text. We represent each word 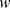 by a vector of unit length, 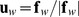, where 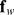 is a vector of dimension 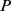 whose components coincide with the frequencies of occurrence of word 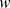 in the different parts, and 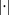 is the 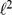-norm. Then, we constructed a similarity matrix 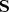 whose elements are defined as 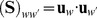. Given the normalization of the vectors 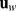, the scalar product 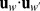 equals the cosine of the angle between the vectors. Since all the vector components are positive, the minimum possible value for the matrix coefficients is zero. However, the similarity coefficient between two words would be zero only when these words are never simultaneously used in any contextual domain of the text, which is statistically rare. Thus, the similarity matrix generally contains non-zero elements for all pairs of words, with higher values for more similar frequency profiles. The connections shown in Figure 2 correspond to the 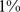 largest elements.

To verify that all these connections were statistically significant, we repeatedly evaluated the similarity matrix on randomized frequency vectors, in which the indices corresponding to the partitions of the text were shuffled independently for each vector. The procedure allowed us to compute a p-value for every connection, given by the fraction of times the connection of the randomised realisation was equal to or higher than that from the original text. As an example, Figure S2 shows some of the resulting semantic networks when method was applied to *On the Origin of Species* by Charles Darwin, using the 400 most informative words.

A similar analysis can be applied to assess the similarity between sections of the text. In this case, a section 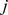 is represented by a unit vector 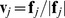, where now the vectors 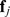 represent the frequencies of the most informative words in section 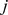. Proceeding in a similar fashion as above a similarity matrix is constructed and its largest elements are selected to define a network linking the sections.

## Supporting Information

Figure S1 **Entropy of individual words in the Voynich Manuscript.** The black circles indicate the entropy of words in the Voynich text as a function of their respective frequency, as computed with Eq. 3. Grey circles show the entropies of words after a random shuffling of all words’ position within the text. The black line corresponds to the analytical average of the entropies for the shuffled text taken over an infinite number of realisations of the shuffling.
(TIFF)

Figure S2 **Semantic networks from** ***On the Origin of Species*** **.** Examples of semantic networks obtained by analysing the co-occurrence patterns of the most informative words in the text, using the word-space method applied to the Voynich manuscript.
(TIFF)

Table S1 **Similarity coefficients between word pairs in the semantic networks shown in** **Figure 2** **.**
(DOCX)

Table S2 **Similarity coefficients between the “thematic” sections of the Voynich manuscript.**
(DOCX)

Table S3 **Most informative words for three books in English.** The words of each source are ranked according to their contribution to the overall information in the distribution of words. The books are the following: *On the Origin of Species*, by Charles Darwin; *The Analysis of the Mind*, by Bertrand Russell; and *Opticks*, by Isaac Newton. The books were downloaded from the Project Gutenberg (www.gutenberg.org).
(DOCX)
