# Supplementary figures and images for: Keywords and Co-Occurrence Patterns in the Voynich Manuscript: An Information-Theoretic Analysis
Source: PLoS One. 2013 Jun 21;8(6):e66344. doi: 10.1371/journal.pone.0066344 (PMC3689824; doi:10.1371/journal.pone.0066344)

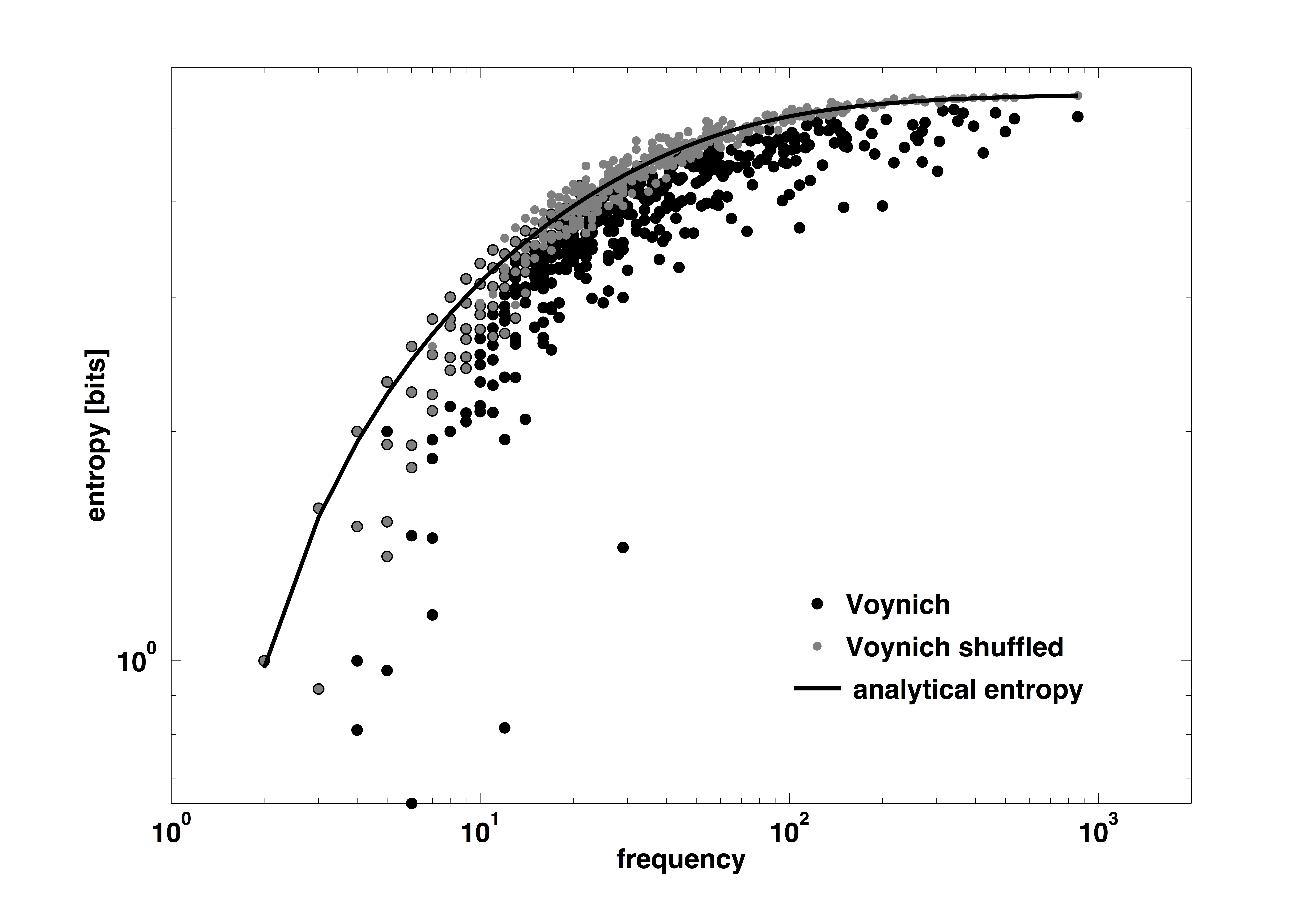

Supplement: Figure S1 — Entropy of individual words in the Voynich Manuscript. The black circles indicate the entropy of words in the Voynich text as a function of their respective frequency, as computed with Eq. 3. Grey circles show the entropies of words after a random shuffling of all words’ position within the text. The black line corresponds to the analytical average of the entropies for the shuffled text taken over an infinite number of realisations of the shuffling. (TIFF) [file pone.0066344.s001.tiff]

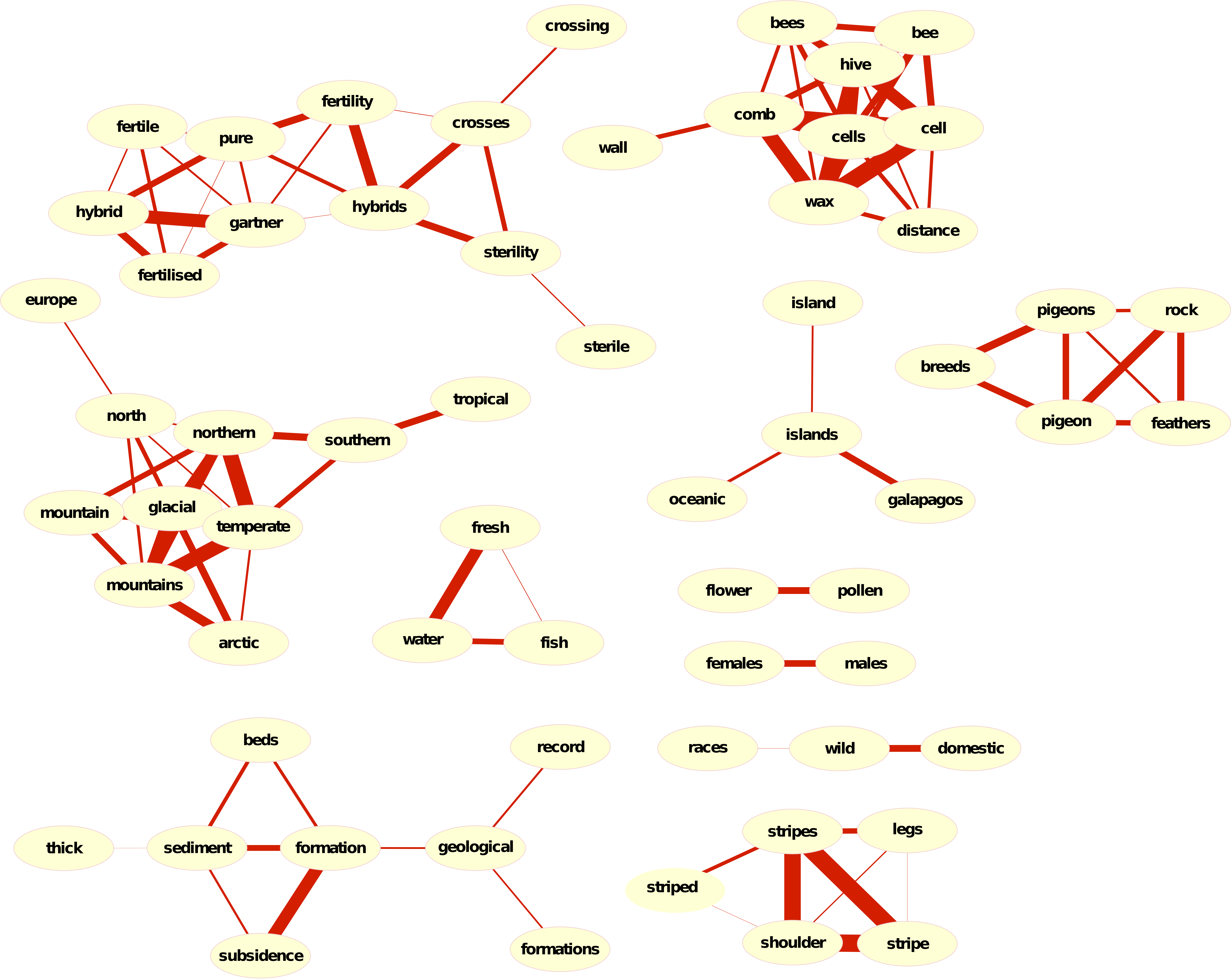

Supplement: Figure S2 — Semantic networks from On the Origin of Species . Examples of semantic networks obtained by analysing the co-occurrence patterns of the most informative words in the text, using the word-space method applied to the Voynich manuscript. (TIFF) [file pone.0066344.s002.tiff]
