# Supplementary material for: Keywords and Co-Occurrence Patterns in the Voynich Manuscript: An Information-Theoretic Analysis
Source: PLoS One. 2013 Jun 21;8(6):e66344. doi: 10.1371/journal.pone.0066344 (PMC3689824; doi:10.1371/journal.pone.0066344)
Supplement: Table S1 — Similarity coefficients between word pairs in the semantic networks shown in Figure 2 . (DOCX) [file pone.0066344.s003.docx]

| **Word pair** | | | **Similarity** |  | **Word pair** | | **Similarity** |
| --- | --- | --- | --- | --- | --- | --- | --- |
| (Cluster A) | | |  |  | (Cluster D) | |  |
| *shedy* | | *chedy* | 0.901 |  | *cthor* | *qotchy* | 0.852 |
| *shedy* | | *qokal* | 0.892 |  | (Cluster E) | |  |
| *qokeedy* | | *qoteedy* | 0.891 |  | *ar* | *aiin* | 0.879 |
| *qokeedy* | | *lchedy* | 0.886 |  | *al* | *ar* | 0.856 |
| *lchedy* | | *qoteedy* | 0.869 |  | (Cluster F) | |  |
| *shedy* | | *qokedy* | 0.869 |  | *r* | *o* | 0.856 |
| *chedy* | | *lchedy* | 0.864 |  | (Cluster G) | |  |
| *qokeedy* | | *chedy* | 0.861 |  | *lkeedy* | *lkeey* | 0.847 |
| *chedy* | | *qoteedy* | 0.860 |  | *lkeey* | *lkaijn* | 0.831 |
| *chedy* | | *qotedy* | 0.855 |  | (Cluster H) | |  |
| *qokal* | | *qol* | 0.852 |  | *ytaiin* | *ykaiin* | 0.848 |
| *chedy* | | *sheedy* | 0.850 |  | (Cluster I) | |  |
| *qokedy* | | *qol* | 0.849 |  | *dar* | *or* | 0.830 |
| *shedy* | | *qol* | 0.848 |  |  |  |  |
| *chedy* | | *checkhy* | 0.847 |  |  |  |  |
| *chedy* | | *qokedy* | 0.843 |  |  |  |  |
| *shedy* | | *qotedy* | 0.840 |  |  |  |  |
| *qokeedy* | | *qokeey* | 0.836 |  |  |  |  |
| *shedy* | | *lchedy* | 0.834 |  |  |  |  |
| *qokain* | | *qol* | 0.831 |  |  |  |  |
| *shedy* | | *sheedy* | 0.831 |  |  |  |  |
| (Cluster B) | | |  |  |  |  |  |
| *okeol* | *qokeol* | | 0.871 |  |  |  |  |
| (Cluster C) | | |  |  |  |  |  |
| *chor* | *chol* | | 0.902 |  |  |  |  |
| *chy* | *shy* | | 0.888 |  |  |  |  |
| *sho* | *chy* | | 0.879 |  |  |  |  |
| *chol* | *sho* | | 0.874 |  |  |  |  |
| *chy* | *shor* | | 0.867 |  |  |  |  |
| *chor* | *shor* | | 0.850 |  |  |  |  |
| *chor* | *cthol* | | 0.849 |  |  |  |  |
| *daiin* | *chol* | | 0.845 |  |  |  |  |
| *chol* | *shol* | | 0.844 |  |  |  |  |
| *dy* | *chy* | | 0.843 |  |  |  |  |
| *cthy* | *shor* | | 0.842 |  |  |  |  |
| *chor* | *sho* | | 0.841 |  |  |  |  |
| *sho* | *cthol* | | 0.841 |  |  |  |  |
| *chol* | *dor* | | 0.838 |  |  |  |  |
