# Supplementary material for: Keywords and Co-Occurrence Patterns in the Voynich Manuscript: An Information-Theoretic Analysis
Source: PLoS One. 2013 Jun 21;8(6):e66344. doi: 10.1371/journal.pone.0066344 (PMC3689824; doi:10.1371/journal.pone.0066344)
Supplement: Table S2 — Similarity coefficients between the “thematic” sections of the Voynich manuscript. (DOCX) [file pone.0066344.s004.docx]

| **“Thematic” Sections** | | **Similarity** |
| --- | --- | --- |
| *herbal* | - *pharma* | 0.834 |
| - *astro* | - *recipes* | 0.789 |
| - *biol* | - *recipes* | 0.760 |
| - *herbal* | - *astro* | 0.675 |
| *astro* | *pharma* | 0.656 |
