# Supplementary material for: Keywords and Co-Occurrence Patterns in the Voynich Manuscript: An Information-Theoretic Analysis
Source: PLoS One. 2013 Jun 21;8(6):e66344. doi: 10.1371/journal.pone.0066344 (PMC3689824; doi:10.1371/journal.pone.0066344)
Supplement: Table S3 — Most informative words for three books in English. The words of each source are ranked according to their contribution to the overall information in the distribution of words. The books are the following: On the Origin of Species, by Charles Darwin; The Analysis of the Mind, by Bertrand Russell; and Opticks, by Isaac Newton. The books were downloaded from the Project Gutenberg (www.gutenberg.org). (DOCX) [file pone.0066344.s005.docx]

| **On the Origin of Species** | **The Analysis of the Mind** | **Opticks** |
| --- | --- | --- |
| *on* | *image* | *rings* |
| *species* | *memory* | *colours* |
| *varieties* | *images* | *prism* |
| *hybrids* | *word* | *paper* |
| *forms* | *belief* | *the* |
| *islands* | *words* | *red* |
| *of* | *desire* | *light* |
| *will* | *sensations* | *I* |
| *selection* | *you* | *rays* |
| *genera* | *past* | *glass* |
| *plants* | *knowledge* | *bodies* |
| *seeds* | *box* | *colour* |
| *sterility* | *content* | *image* |
| *fertility* | *consciousness* | *was* |
| *characters* | *appearances* | *blue* |
| *breeds* | *movements* | *refraction* |
| *groups* | *mnemic* | *water* |
| *water* | *feelings* | *greek* |
| *the* | *proposition* | *lens* |
| *formations* | *general* | *white* |
